# Vertical Transmission of *Babesia microti* in BALB/c Mice: Preliminary Report

**DOI:** 10.1371/journal.pone.0137731

**Published:** 2015-09-15

**Authors:** Malgorzata Bednarska, Anna Bajer, Anna Drozdowska, Ewa J. Mierzejewska, Katarzyna Tolkacz, Renata Welc-Falęciak

**Affiliations:** Department of Parasitology, Institute of Zoology, Faculty of Biology, University of Warsaw, 1 Miecznikowa Street, Warsaw, Poland; Albert Einstein College of Medicine, UNITED STATES

## Abstract

*Babesia* spp. (Apicomplexa, Piroplasmida) are obligate parasites of many species of mammals, causing a malaria-like infection- babesiosis. Three routes of *Babesia* infection have been recognized to date. The main route is by a tick bite, the second is via blood transfusion. The third, vertical route of infection is poorly recognized and understood. Our study focused on vertical transmission of *B*. *microti* in a well-established mouse model. We assessed the success of this route of infection in BALB/c mice with acute and chronic infections of *B*. *microti*. In experimental groups, females were mated on the 1^st^ day of *Babesia* infection (Group G0); on the 28^th^ day post infection (dpi) in the post- acute phase of the parasite infection (G28); and on the 90^th^ and 150^th^ dpi (G90 and G150 group, respectively), in the chronic phase of the parasite infection. Pups were obtained from 58% of females mated in the post-acute phase (G28) and from 33% of females in groups G90 and G150. Mice mated in the pre-acute phase of infection (G0) did not deliver pups. Congenital *B*. *microti* infections were detected by PCR amplification of *Babesia* 18S rDNA in almost all pups (96%) from the experimental groups G28, G90 and G150. Parasitaemia in the F1 generation was low and varied between 0.01–0.001%. Vertical transmission of *B*. *microti* was demonstrated for the first time in BALB/c mice.

## Introduction

Vertical transmission is a phenomenon observed in many parasite and host species. Vertical transmission occurs by the transfer of the parasite from mother to offspring during pregnancy (*Toxoplasma gondii*), child birth (*Trichomonas vaginalis*) or lactation (*Toxocara canis*) [1–4]. Congenital infections may cause complications during pregnancy, miscarriages or foetal infections [5,6]. Vertical transmission of parasites may have a negative impact on ontogenesis and result in inborn defects in offspring. Vertical transmission has been reported for protozoa of the genus *Plasmodium*, *Toxoplasma*, *Trypanosoma* and for nematodes of the genus *Toxocara*, *Ascaris* and *Strongyloides* [1–4,7–10].

Congenital infection has been recently reported in parasites of the genus *Babesia* (Apicomplexa, Piroplasmida) [6,11–16]. Protozoa of the genus *Babesia* are obligate parasites of many mammalian species. They cause babesiosis, a malaria-like infection characterised by erythrocyte invasion and destruction, which can be life-threatening in immunocompromised humans [17–21]. There are three routes of transmission of this parasite [22]. The main route of infection with *Babesia* spp. is by a tick bite. Parasites can also be transferred via blood transfusion or direct contact with the blood of infected hosts [23]. The third, vertical route of infection is very poorly recognised and understood [6,11–16]

Transmission of *Babesia* spp. from a female to her offspring has been reported in mammals: in bovines (*B*. *divergens*), sheep (*B*. *ovis*), dogs (*B*. *gibsoni*) and horses (*B*. *caballi*, *B*. *equi*) [6,16,22,24–26]. In Poland, the first cases of vertical transmission of *B*. *canis* in dogs were described by Mierzejewska et al. [14]. There are also several reports on congenital *B*. *microti* infections in humans [12,27–32]. In most of the aforementioned human cases, babesiosis manifested in children several weeks after birth. Infants had flu-like symptoms, anaemia and hepatomegaly. Serological examinations of mothers and children revealed positive results for *B*. *microti* antigens. To date, congenital infections with *B*. *microti* have been reported only occasionally in humans, but the factors affecting the risk of its occurrence (i.e. frequency/ success rate; phase of infection) are not known. To date, no systematic research has been conducted on the vertical transmission of *B*. *microti*. To the best of our knowledge, this is the first study on this route of transmission in well-controlled laboratory conditions on BALB/c mice. The main aim of these experiments was to determine the occurrence and the success of vertical transmission in females with acute and chronic infections of *B*. *microti*.

## Materials and Methods

### Animals

This study was carried out in strict accordance with the recommendations in the Guide for the Care and Use of Laboratory Animals of National Ethics Committee for Animal Experimentation. The protocols no 233/2011 and 406/2013 were approved by First Warsaw Local Ethics Committee for Animal Experimentation.

There were 155 mice of the BALB/c strain involved in the experiments. This number consisted of 51 females, 15 sire males aged 10–12 weeks (supplied by Animal Facility, Faculty of Biology, University of Warsaw) and 89 pups (41 females and 48 males), aged from birth to 26 weeks old at the end of experiment. All mice were housed in plastic cages with sawdust. Females infected with *B*. *microti* before mating or not mated (control) were housed in groups of five/six. Pregnant females and dams with their offspring were housed individually. The sire males of BALB/c strain were also housed individually whilst not paired with females.

### Parasites

*B*. *microti* strain King’s 67 was originally obtained from Professor Sarah Randolph (University of Oxford, UK) in 1997. The parasite is maintained in our department in BALB/c mice by carrying out blood passages using intraperitoneal (i.p.) injections every two weeks. In our experiments, mice were infected (i.p.) with 5 x 10^6^ *B*. *microti*- infected red blood cells to the volume of 0.2 ml per mouse. This method of *Babesia* transfer and the dose of infected erythrocytes have been successfully used in experimental studies on *Babesia* and other blood parasites worldwide [33–35]. The course of experimental infection of *B*. *microti* King’s 67 strain in laboratory mice has been studied in our department for many years. Acute and chronic phases of the infection have been determined and compared between the different strains of mice, from sensitive (CBA/2), through intermediate-sensitive (BALB/c) to resistant strains (DBA/2, C57BL6) (data not published). BALB/c mice were found to be the most useful model for experimental studies.

### Mating and Breeding of Mice

Males were introduced to the home cages of single-housed females and kept together for 7 days. Females were housed individually during pregnancy (19–21 days; mean length of pregnancy period) or with their pups. Pups were housed with their mothers until weaning (28th day of life). Then pups were sexed and males and females were kept separately.

### Experimental Design

Each experimental group consisted of 3–6 females:

#### Group G0

Females expected to become pregnant during the **acute phase** of the *B*. *microti* infection. Females were paired with males on the day of infection with *B*. *microti*. We performed three repetitions on 18 females in total.

#### Group G28

Females expected to become pregnant in the **post-acute phase** of the *B*. *microti* infection. Females were mated on the 28^th^ dpi with *B*. *microti*. We performed two repetitions on 12 females in total.

#### Groups G90 and G150

Females expected to become pregnant in the chronic phase of the *B*. *microti* infection. Females were mated on the 90^th^ dpi or the 150^th^ dpi with *B*. *microti*, respectively. We performed two independent experiments for the study in the **chronic phase** (12 females in total).

Control groups:

#### Gcontrol

6 females were infected with *B*. *microti* to monitor the course of the *B*. *microti* infection in the absence of pregnancy.

#### Geryt

3 females receiving intraperitoneal administration of uninfected red blood cells (RBCs). Females were paired with males on the day of injection with uninfected RBCs.

Pregnancy was confirmed by the observation of a vaginal plug and an increase in the body size of the females. From the 19^th^ day of pregnancy, pregnant females were observed daily; in addition the number of pups in the litter at delivery was recorded.

### Detection of *B*. *microti* Infection

The course of *B*.*microti* infection was monitored by two methods: detection of parasitaemia and PCR technique. Determination of parasitaemia (% of infected red blood cells [iRBC]) was used to monitor the dynamic changes during the acute phase of infection (until 28^th^ dpi) in stained blood smears in all experimental groups (G0, G28, G90, G150, Gcontrol). Smears were made from a drop of blood from the dorsal tail vein of an infected mouse. The infected erythrocytes were visible in blood smears as early as 5–6 hours post infection. To monitor the possible re-occurrence of the acute phase, blood samples were collected individually every two weeks until the end of experiment. Additionally, blood samples were collected every 3–4 days in the experimental groups during pregnancy and the lactation period. To detect the chronic phase of the infection by PCR (not detectable on blood smears), blood samples were collected every 1–2 weeks from the 28^th^ dpi, until the end of the experiment.

In our model, the **acute phase** of the infection occurs from 4 to 12 days post infection with *B*. *microti* and manifests with high parasitaemia (between 40 and 70% iRBC); **post- acute phase** of infection is a period of continuous decline in parasitemia up to a few percent (3–5 weeks post-infections). **Chronic phase** of infection is characterised by very low or undetectable parasitaemia, but infection is detectable by PCR.

### Microscopic Examination

Blood smears were air dried, fixed in 96% methanol, stained with Diff-Quik staining kit (Medion Diagnostics AG, Switzerland) and examined under oil immersion (x1000 magnification) for parasitaemia estimation. A total of 20 or 200/300 fields during the acute or chronic phase of infection, respectively, were scanned by two people independently and the number of iRBC was recorded. Parasitaemia was expressed as the % of infected cells.

### Detection of *B*. *microti* DNA by PCR

Blood samples for molecular diagnosis were collected into 0.001M EDTA and frozen at a temperature of -20°C until DNA extraction. DNA extractions were performed on whole blood using the AxyGen MiniPrep Blood kit (AxyGen, USA). The extracted DNA was subjected to PCR with specific primers for *Babesia* spp. small subunit ribosomal RNA (*18S rRNA*) in conditions previously described [36]. Primers: BABGF2 (5’-GYYTTGTAATTGGAATGATGG-3’), BABGR2 (5’-CCAAAGACTTTGATTTCTCTC-3’) were used for the amplification of the 559 bp gene fragment. The resulting PCR products were separated by electrophoresis in 1.5% agarose gel.

We followed standardised protocols to avoid contamination of samples, i.e. DNAs were extracted from blood samples using sterile plastics on three separate occasions, negative and positive controls were implemented in each set of PCRs, PCR reactions were repeated at least twice to confirm a positive or negative result.

### Identification of Congenital Infection

The occurrence of congenital infection was evaluated in all pups. For microscopic study blood samples from F1 mice were taken individually from the dorsal tail vein, every 1–3 weeks from the 25^th^ to the 120^th^ day post born (dpb). For the PCR method [31], blood samples were collected every 1–3 weeks from the 25^th^ until the end of the experiment (180 dpb). Blood samples from the pups were collected since the 25^th^ dpb because of the small size of the F1 generation in the first three weeks of life. Since the day of birth until the 25^th^ dpb, the general health of the pups was observed daily. We didn’t observe any symptoms of malaise, weakness or other symptoms of the disease that point to an acute course of the infection.

### Statistical Analysis

Fisher’s exact test (Instat software) was used to compare the reproductive success (% of females that delivered pups) between experimental groups. ANOVA (SPSS) was used for the comparison of the courses of infection (parasiteamia) in females and their offspring.

## Results

### The Course of *B*. *microti* Infection in Different Groups of Females

**Fig 1:**
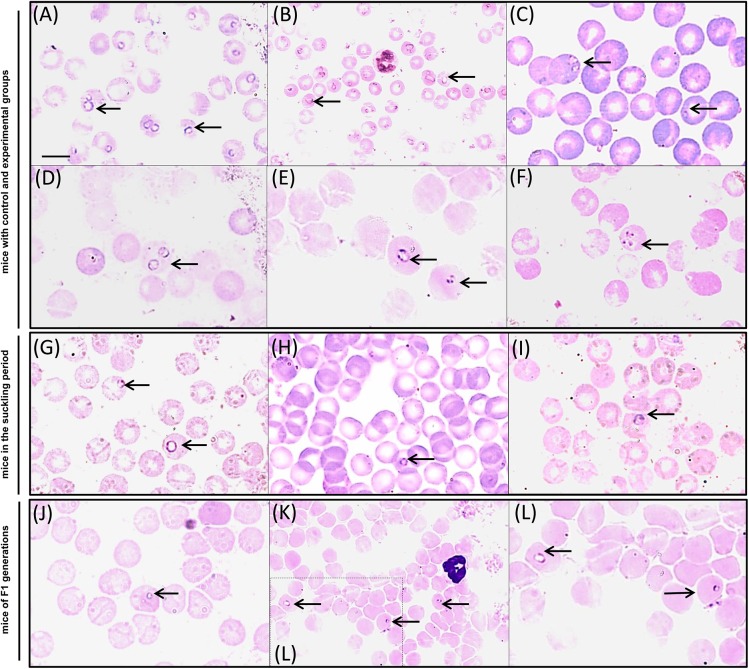
Peripheral blood smears from experimental BALB/c mice and their offspring demonstrating parasitised erythrocytes in different days post infections with *B*. *microti*. Microscopic images of infected erythrocytes under light microscopy of blood smears stained with Giemsa stain. **(A-F)** blood smears from mice with control and experimental G0, G28, G90, G150 groups. Pictures A and B showing pleomorphic rings and multiply-infected rbcs in the acute phase of the infection in: (A) a mouse (no 282) from group G28 on 5 dpi and (B) a mouse (no 1) from group Gcontrol on 7 dpi. Pictures C-D showing infected erythrocytes with single rings of *B*. *microti* in post-acute and chronic phase of infection in: (C) a mouse (no 285) from group G28 on 18 dpi, (D) a mouse (no 906) from group G90 on 28 dpi. Picture E demonstrates the divided form in the chronic phase of the infection in a mouse (no 1504) from group G150 on 97 dpi. Picture F demonstrates the ‘Maltese cross form’ in a mouse (no 904) with post-acute infection from group G90 on 28 dpi. **(G-I)** blood smears from mice in the suckling period. Pictures G-I showing infected erythrocytes (in the suckling period) of mice pregnant in the post-acute phase of the parasite infection in: (G) mouse no 281 from group G28 on the first day post weaned (49dpi), (H) mouse no 284 from group G28 on the 3rd day post weaned (51dpi), (I) mouse no 286 from group G28 on the 6th day post weaned (56 dpi). The course of infection with *B*. *microti* in these three mice is presented on Fig 4. **(J-L)** blood smears from mice of F1 generations. Pictures J-L showing infected erythrocytes in BALB/c pups vertically infected with *B*. *microti* in (J) mouse no 15012 on 50 dpb born from mother from the G150 group, (K-L) mouse no 1504 on 97 dpb born from mother from the G150 group, (L) the enlarged fragment of the image K. Scale bar represents 10 μm. Magnification x 1000 for A, C-J pictures and x600 for B, J-K pictures. Black arrows show infected erythrocytes.

**Fig 2:**
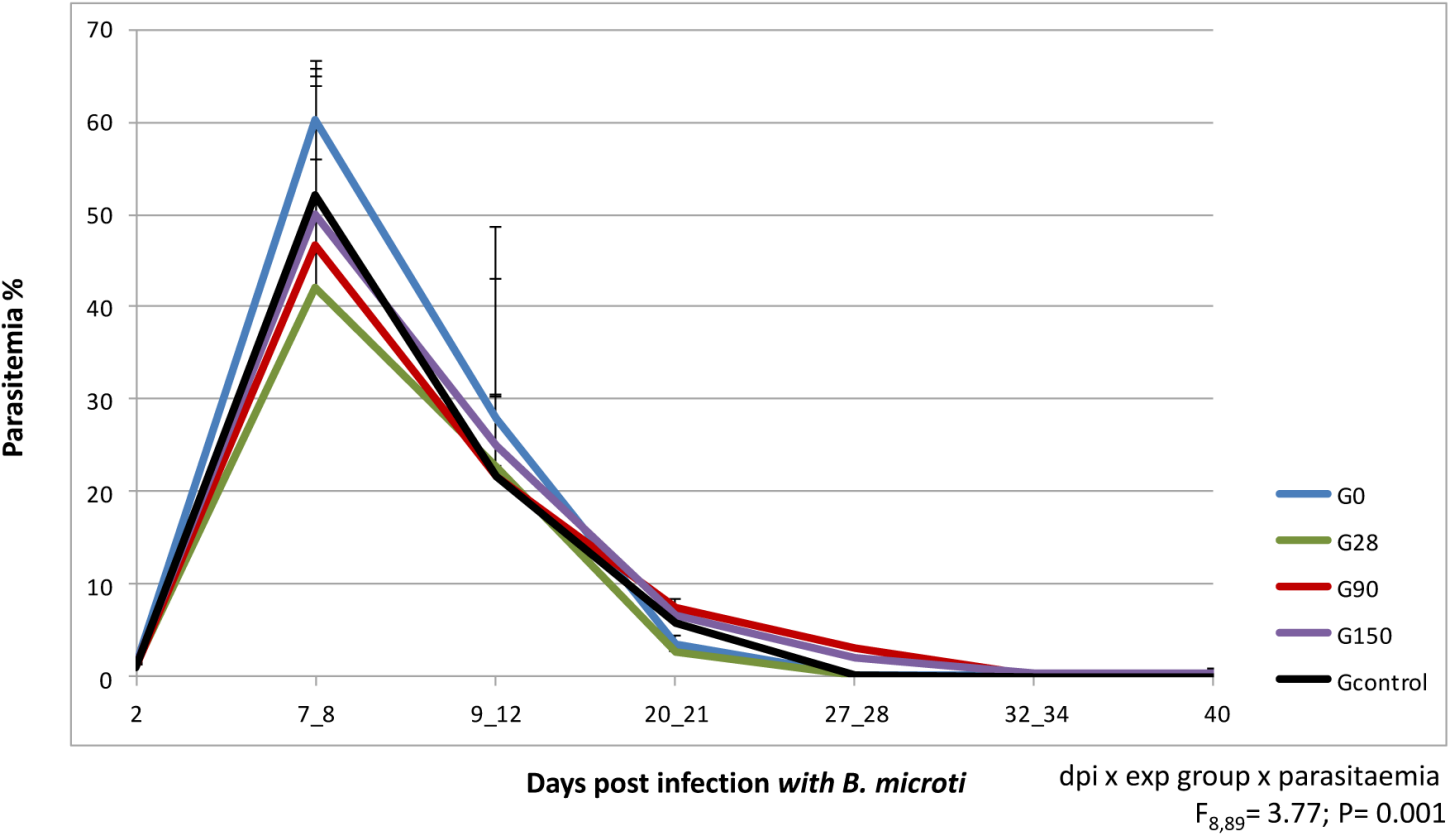
Comparison of the course of *B*. *microti* infection in mice pregnant in different phases of the parasite infection. Level of *B*. *microti* parasitemia on the selected day post infection. Parasitemia is shown as a percentage of infected erythrocytes found in mouse peripheral blood, measured on the Giemsa-stained thin smears. Mice from the experimental groups were infected intraperitoneally with 5 x 10^6^ *B*. *microti* iRBCs. Datum points are the mean for six animals. The experimental groups are marked: Blue line- females paired with males on the day of infection (G0); Green line- females paired with males on the 28^th^ day of infection (G28); Red line- females paired with males on the 90^th^ day of infection (G90); Violet line- females paired with males on the 150^th^ day of infection (G150). Black line- control female infected with *B*. *microti*, not mated. Each time point represents the mean value ± one SD. on the graphs.

Parasites were observed in erythrocytes of almost all animals on the second dpi. Parasitised erythrocytes in blood smears of BALB/c mice from experimental groups on different days post infection with *B*. *microti* are presented in Fig 1A–1F. One female in the G90 group was positive from the 9^th^ dpi, with the highest parasitaemia on the 16^th^ dpi (12.7%). A comparison of the courses of *B*. *microti* infection during the acute and post-acute phases of infection (on days 7–8, 9–12, 20–21) in the experimental groups is presented in Fig 2. The typical course of infection was observed in all groups (dpi x parasitaemia: F_2, 89_ = 88.8; P< 0.001), with an acute phase marked by high parasitemia during the first two weeks and a chronic phase with low levels of parasitemia that followed. Significant differences in mean parasitemia between the experimental groups were observed (exp group x parasitaemia: F_4, 89_ = 3.94; P = 0.006) and finally, significant differences in the course of parasitaemia were observed between the groups (dpi x exp group x parasitaemia F_8,89_ = 3.77; P = 0.001) (Fig 2). Mean parasitaemia in the acute and post-acute phases of infection was the highest and similar (30–31%) in Gcontrol and G0 groups and in the acute phase of infection the highest parasitaemia (above 60%) were observed in mice of G0 group, mated on the day of infection with *B*. *microti*. Parasitaemia on the 7–8 dpi in all other experimental groups was below the mean parasitaemia in G0 group (Fig 2). In all mice parasitaemia was significantly lower on the 9^th^-12^th^ dpi (21–27%) and then decreased below 0.5% or was undetectable, on the basis of microscopical observations, from the 40^th^ dpi.

**Fig 3:**
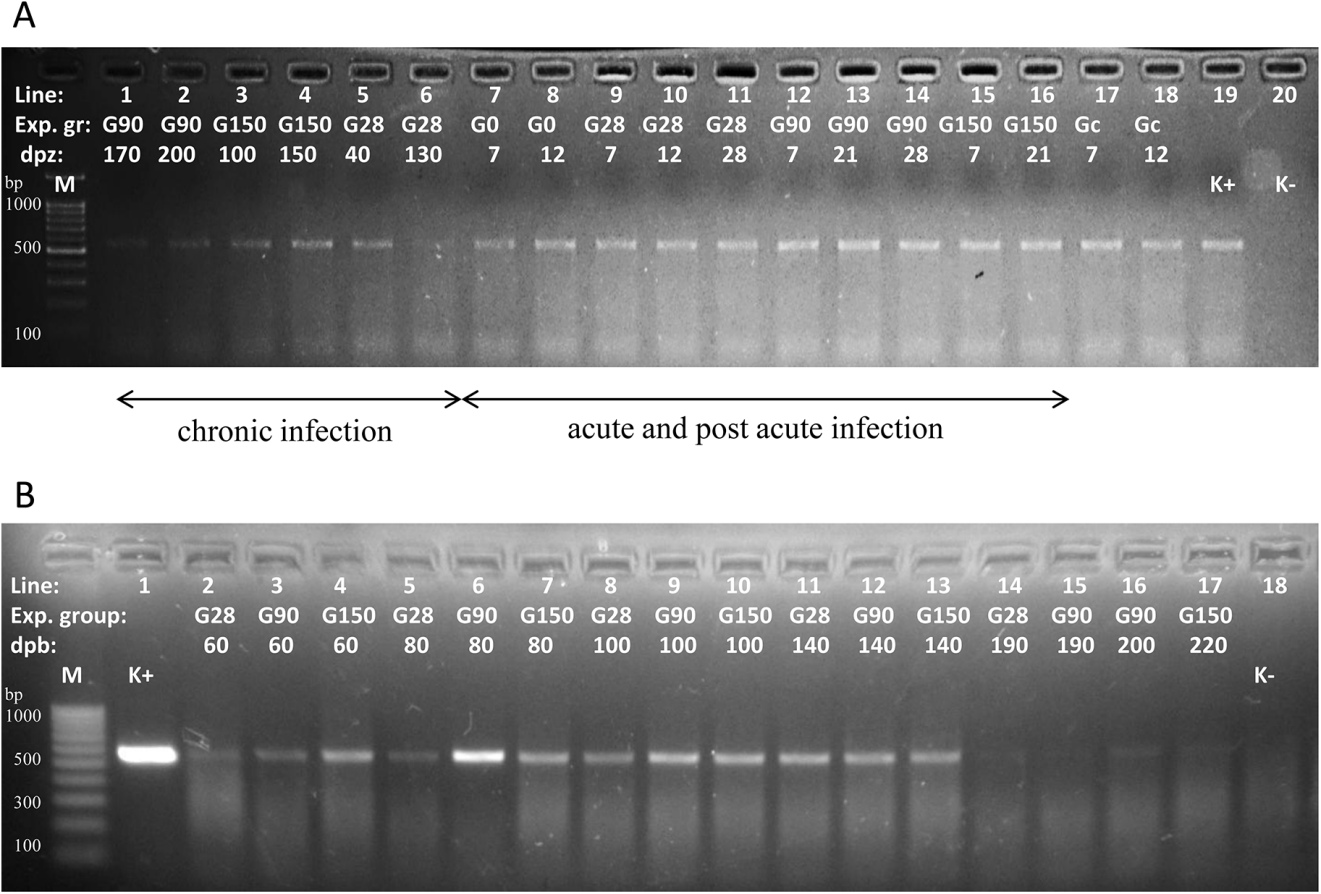
PCR products from *B*. *microti* infected BALB/c mice on Midori Green stained gel. **(A)** PCR products from experimental infected BALB/c mice on a different day post infection with *B*. *microti*. Lane 1–6 mice chronically infected with G90, G150, and G28 group, respectively; Lane 7–16 mice with acute and post-acute infection with G0, G28, G90, G150, and Gcontrol, respectively; Lane 19 –positive control, Line 20 –negative control; dpi- day post infection with *B*. *microti*; (B) PCR products from vertically infected BALB/c mice of the F1 generation. Lane 1 –positive control, Line 18 –negative control; Lane 2–4 mice on 60 dpb born from a mother within the G28, G90, G150 group, respectively. Lane 5–7 mice on 80 dpb born from a mother within the G28, G90, G150 group, respectively. Lane 8–10 mice on 100 dpb born from a mother within the G28, G90, G150 group, respectively. Lane 11–13 mice on 140 dpb born from a mother within the G28, G90, G150 group, respectively. Lane 14–15 mice on 190 dpb born from a mother within the G28 and G90 group respectively; M-marker site, 100bp DNA ladder; dpb- day post born; Gc- Gcontrol.

Chronic infections with *B*. *microti* were confirmed by PCR amplification of *Babesia* DNA from the 40^th^ to 180^th^ dpi in all females in groups G28 and G90. In group G150 females were mated on the 150^th^ dpi and the course of infection was monitored up to the 230^th^ dpi (60 days post-delivery). Mice from groups G0, G28, G90, and G150 were positive by PCR until the end of experiment. PCR products from *B*. *microti* infected BALB/c dams from experimental groups are presented on Fig 3A.

### The Course of *B*. *microti* Infection in Pregnant and Lactating Females

The reactivation of infection, determined by the increase of the parasitaemia, was observed during pregnancy and lactation in mice from groups G28 and G150. In this group, eight of the 12 females delivered offspring. In females that became pregnant, parasitaemia was very low but detectable by microscopy in blood smears (overall 0.53%, in a range of 0.06–1.4%) (Fig 1G–1I. Parasitaemia was detectable until the end of lactation (20 dpb). In the remaining females (not pregnant) parasitaemia was undetectable from the 32^nd^ dpi to the end of the experiment.

**Fig 4:**
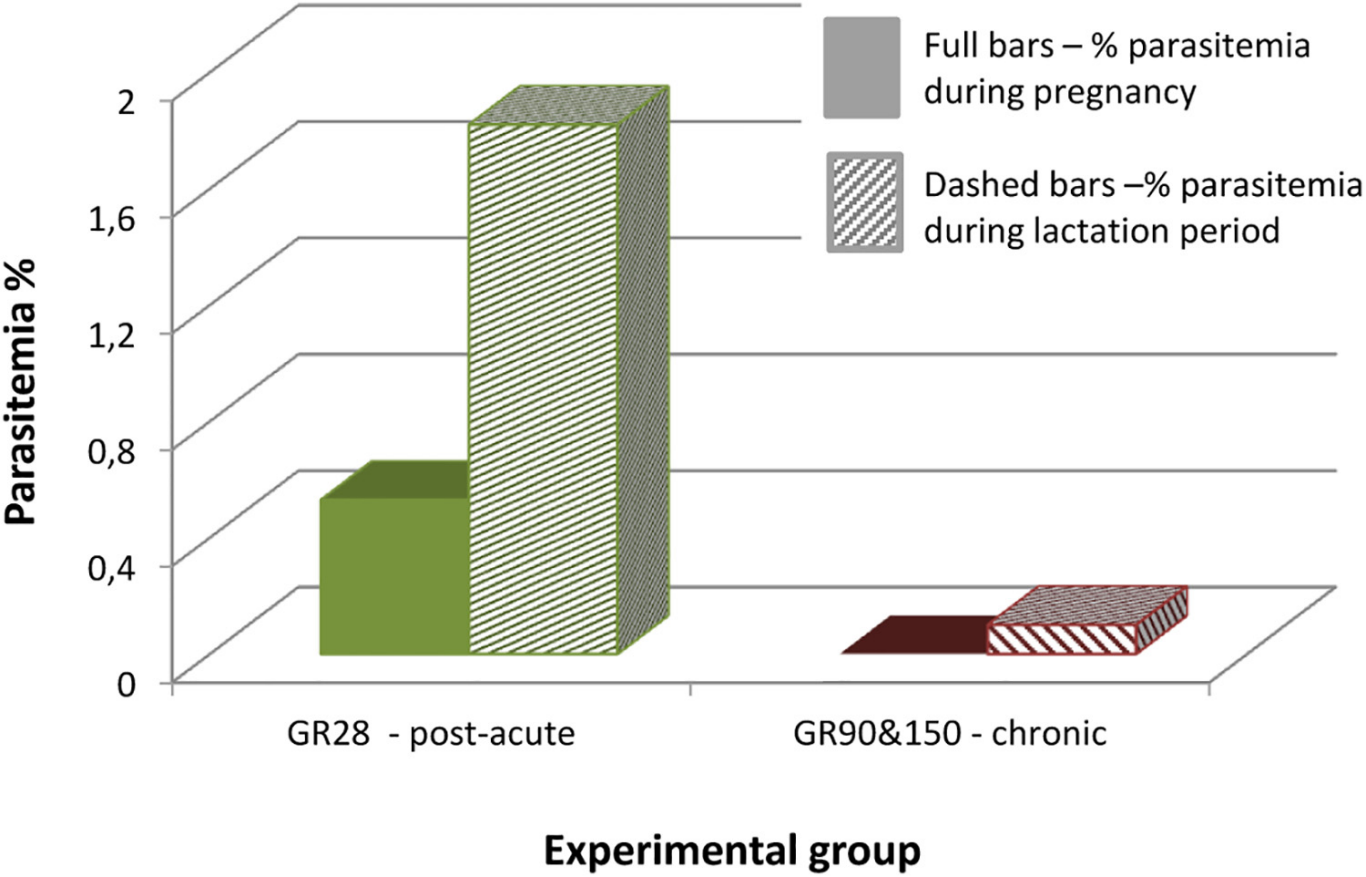
Comparison of the overall parasitaemia during pregnancy and lactation, in females mated on post–acute phase on the 28^th^ and on chronic phase on the 90^th^ and 150^th^ day post infection with *B*. *microti*. Experimental groups are marked: Green bars- females paired with males on the 28^th^ day of infection (G28); Red bars- females paired with males on the 90^th^ and 150^th^ day of infection (G90 and 150).

A different pattern emerged in groups G90 and G150 compared to group G28 (due to the lack of differences between females of G90 and G150, results are presented together on Fig 4). Several mice with previously negative blood smears developed low level parasitemia during pregnancy (0.53%) and lactation (1.82%). Detectable parasitemia during pregnancy and lactation was noted in all G28 mice (n = 7) who delivered/nursed, and detectable parasitemia during the lactation phase only was noted in the two of G150 mice who delivered/nursed. There was no detectable parasitemia during pregnancy/lactation in any of the G90 mice, or during the same time period for G28 or G150 mice that did not become pregnant. Although these differences were not significant, there was clear trend presented in Fig 4.

### Reproductive Success of BALB/c Mice Infected with *B*. *microti*

The vaginal mucus plug was detected in all females on the second or third day after joining with males. Births were reported in dams from three experimental groups and the control Geryt group. Differences in reproductive success (mean number of pups/ litter) between the experimental groups were significant (Tab 1). Litters were obtained from 58% (7/12) of females infected in the post-acute phase (group G28) (Tab 1). Births were reported in 8 females from this group but litters were obtained from only 7 females. One female had eaten pups immediately after birth. In this female, four scars were detected on the uterus during autopsy. No mortality was observed in the other offspring. Litters in the other experimental groups were obtained from 33% (4/12) of females in the chronic phase of the *B*. *microti* infection (in both groups G90 and G150). Females mated in the pre-acute phase of the infection (group G0) did not deliver any pups in three independent experiments.

Births were also reported in all females mated on the day of the injection with uninfected RBCs (group G0eryt). The mean number of pups in a litter was 8 (in range 7–9 pups/ litter). No mortality was observed in this offspring.

### Vertical Transmission

The DNA of *B*. *microti* was detected in blood samples (collected from 20 dpb to 220 dpb) of 62 pups from infected females, thus confirming the occurrence of congenital infection (success of vertical transmission 96%). The details of the congenital infection in the experimental groups are shown in Tables 1 and 2. In total, parasites were observed in blood smears in 57% (37/65) of pups (F1) delivered by infected females (Tab 1). Parasitaemia was very low and the overall rate of iRBC was 0.005% (Tab 2). There were no symptoms of the acute phase of the *Babesia* infection (dark-colored urine, febrile seizures, anorexia, apathy) observed in pups. Parasitised erythrocytes in blood smears and PCR products from vertically infected BALB/c pups are presented on Fig 1J–1L) and 3B, respectively.

**Table 1:** Comparison of the reproductive success and features of congenital *B*. *microti* infection in pups of females from experimental groups.

Experimental group /phase	G0/	G28/	G90 &G150/	Statistical
of infection	acute	post-acute	chronic*	values
**Reproductive success (%)**	**0%**	**58%**	**33%**	χ^2^ = 13.2
**No. of mothers/females**	0/18	7**/12	4/12	df = 2 **p = 0.014**
**Total and**	0	40	25	F_2, 17_ = 29.5
**mean no. of pups (+/- SD)**	**0**	**5.71 ±0.60**	**6.25 ±0.80**	**p<0.001**
**and sex ratio in**	ND	19:21	13:12	
**F1 males: females**				
**Symptoms in F1**	asymptomatic	asymptomatic	asymptomatic	N/A
**(until the 120** ^**th**^ **dpb)**				
**No. (%) of pups with detected parasitaemia**	ND	22/40 (55%)	15/25 (60%)	NS
**(no. of infected/tested)**				
**No. (%) of F1**	ND	12: 10	9:6	NS
**male: female**		(63%: 48%)	(60%: 40%)	Fisher’s exact
**with detected parasitaemia**				test: p = 0.21
**No. (%) of PCR positive**	ND	37/40 (92.5%)	25/25 (100%)	NS
**in F1 generation**				
**(no. of infected/tested)**				

* Due to the lack of significant differences between females infected in the chronic phase of infection (G90 and G150) results are presented together.

**Births were reported in 8 dams but litters were obtained only from 7 females. One of the dams had eaten pups immediately after birth. Four scars were observed on the uterus but this data was not incorporated into the analysis.

**Table 2:** The mean parasitaemia (% infected RBC) in pups delivered by a female from an experimental group.

Pups from experimental group	Day post birth						
	25	35	50	60	75	90	120
**F1 of G28**	0.004	0.003	0.002	0	0.001	0	0.004
**n = 17**	(0.0–0.02)	(0.0–0.006)	(0.0–0.008)		(0.0–0.009)		(0.0–0.012)
**F1 of G90**	0.001	0.002	0.003	0.003	0.0004	0	0.0004
**n = 14**	(0.0–0.005)	(0.0–0.006)	(0.0–0.01)	(0.0–0.1)	(0.0–0.002)		(0–0.004)
**F1of G150**	0.007	0.0007	0.004	0.003	0.001	0.0002	0
**n = 11**	(0.0–0.02)	(0.0–0.003)	(0.0–0.01)	(0.0–0.003)	(0.0–0.004)	(0–0.001)	

Data is presented for selected days post birth. In parentheses are the minimum and maximum values of parasitaemia for selected days in the experimental groups.

### The Course of Congenital *B*. *microti* Infection in the F1 Generation

The course of the congenital infection of *B*. *microti* in the F1 generation, born to mice from experimental groups G28, G90, and G150 is presented in Tab 2. Parasitaemia in congenital infections was detectable in blood smears on different days post birth, and occasionally being undetectable, suggesting only a chronic phase of infection. Although no acute phase was observed in the F1 generation, the difference in the course of infection between dams and their pups was not significant, likely due to a long period of the chronic phase observed in both groups of mice.

## Discussion

This study investigated the occurrence and the success of the vertical transmission of *B*. *microti* in the BALB/c mouse model. Our findings suggest that *B*. *microti* can be transmitted to offspring and the success of vertical transmission is dependent on the phase/ intensity of the parasite infection. Moreover we suggest that the acute phase of the infection in breeding females increases the risk of mortality of embryos /foetuses. To our knowledge, this is the first report on the congenital infection of *B*. *microti* in BALB/c mice.

To confirm this hypothesis we examined the possibility of vertical transmission of *B*. *microti* in a laboratory model. In our study, BALB/c females were mated in different phases of the *B*. *microti* infection. All successful females presented very low parasitaemia (0–0.1%) on the day of mating. Analysis of the relationship between the time of mating and the phase of infection (acute, post-acute or chronic infection) showed that the pups were delivered only by mothers with post-acute or chronic infections. Females mated on the day of infection with *B*. *microti* didn’t deliver pups. We only observed some trends (differences NS) in the reproductive success of females (higher in females mated in the post-acute phases of the *B*. *microti* infection than in females mated during a chronic infection with *Babesia*), and did not observe any differences in the litter size. We observed a similar number of pups in the experimental and control groups. It seems that *B*. *microti* infection during pregnancy in BALB/c mice may limit the number of females capable of reproduction, but doesn’t affect the number of animals per litter in successful breeding.

Our experiment showed that *Babesia* infection in experimental mice resulted in unsuccessful breeding in more than half of the infected females. Moreover the mean number of pups/ litter differed significantly between exp groups however, dams age could account for this differences. Healthy, 3- months old, not-infected pregnant BALB/c females usually deliver 4.73 pups in a litter, Animal Facility at the Faculty of Biology, University of Warsaw, and the reproductive success was 97.25% (218/224). The data for older females are similar. Dams in age 5 and 6 months usually deliver 5.1 or 5.32 pups in a litter and the reproductive success was 93% (964/964+66) and 91% (368/368+33) respectively. However, the pathological mechanisms that led to pregnancy loss during a *B*. *microti* infection remain to be determined. Different factors could have contributed to this result. It seems that in females mated on the same day as when infected with *B*. *microti*, the early stages of development/ ontogenesis were affected. The presence of a vaginal plug and observed scars on uterus branches (data not presented) suggested that mice had been fertilized and foetal loss had happened at a very early stage of the pregnancy. Development of an acute parasite infection could have caused early absorption of the embryos. Successful breeding of females treated with an intraperitoneal injection of uninfected RBC (group Geryt) suggested that non-specific immunity could not be the main reason for the lack of offspring in the G0 group. The lack of knowledge about the impact of *Babesia* spp. infection on early stages of pregnancy in mice or other animals needs further study. Infection with another parasite of the Apicomplexa phylum, *Plasmodium chabaudi* had a negative impact on early pregnancy in AS/C57BL/6 mice [2].

Interestingly, the effect of pregnancy on the course of the *Babesia* infection tended to differ depending on the experimental groups. In mice mated during the post-acute phase of the infection the parasitaemia increased again during the pregnancy and lactation periods, but in other groups, in mice with a chronic invasion, this increase was not observed. Further studies are needed to confirm our hypothesis that parasites can modulate the immune system and when chronic infection is established and well stabilised in mice, the hormonal changes associated with the development of pregnancy do not affect the course of the *B*. *microti* infection.

We found that almost all pups delivered by infected females were congenitally infected with *B*. *microti*. The occurrence of the vertical transmission was confirmed using PCR and quantitative techniques (level of parasitaemia). As expected, PCR was more sensitive in detecting congenital infection than microscopical observation of blood smears, demonstrating that 95% of offspring were infected. It seems that the occurrence and intensity of congenital infection may depend on the species of *Babesia*. High success of vertical transmission (100%) was demonstrated in an experimental invasion of *B*. *gibsoni* in a Beagle female, who after two years of chronic infection delivered 5 puppies, all with fatal congenital babesiosis [6]. Less successful was vertical transmission of *B*. *canis* in dogs described by Mierzejewska et al. [14]. The Central Asian Shepherd dog bitch naturally infected with *B*. *canis*, delivered 10 puppies. Three of them (30%) were congenitally infected with *B*. *canis*. All puppies were infected at the same time on the 44^th^ day of life and all developed acute babesiosis. Following treatment and blood transfusion, these pups recovered. In the case of the reported congenital infection of *B*. *bovis* in a calf (babesiosis manifested two days after birth) the treatment was successful and the disease was cured [16]. The epidemiologic implications of *Babesia* spp. vertical transmission in domestic and reservoir hosts are still unknown and should be investigated. Similar differences in the success rate of the vertical transmission, dependent on parasite species, were observed in experimental *Leishmania* infections [37]. Congenital invasions were found in 25.8% of the pups of hamsters infected with *L*. *panamensis* and in only 14.6% of the pups of hamsters infected with *L*. *donovani*.

In the cases of vertical transmission in humans the course of innate *B*. *microti* infection was initially asymptomatic in almost all cases, and between the 26^th^ and 41^st^ day after birth children developed acute babesiosis. In congenital cases of human babesiosis, all children recovered following treatment and blood transfusion. Before pregnancy, only one mother presented symptoms of the disease [12].

In our study we found that parasitaemia was detectable in only 57% of pups. All pups showed ‘limited’ infections in which the number of parasites was maintained constantly at a very low level (below 0.01%). Different factors could have contributed to this result. It might have been caused by not ‘typical trans-placental’ (in opposite to tick bite) transmission of *Babesia*. Additionally, the specific anti-*Babesia* IgG-mediated immune response of mothers with *Babesia* infection can have led to the control of parasitaemia and guarantee a very low level of infected erythrocytes in pups with inborn infections and maternal immunity [38, 39]. Unfortunately, there is still nothing known about the route of *B*. *microti* invasion to the foetus and this problem requires further studies.

Interestingly, we found sex-associated differences in BALB/c mice susceptibility to the *B*. *microti* infection. The infection of adult BALB/c males with three different doses of infected erythrocytes (10^4^–10^6^) was fatal (data not presented). These results are consistent with another study where sex-dependent susceptibility to parasite infections differed in males and females [19, 40–44]. In this study there were no significant differences (only trends) between males and females, among the F1 generation. The reasons for the different courses of *B*. *microti* infection in adult and F1 BALB/c males and females needs further study, as they are likely due to the output of different hormonal statuses or different sex-dependent immunity mechanisms.

In conclusion, this preliminary experimental study shows that *B*. *microti* infection in a BALB/c female could lead to vertical transmission, resulting in an asymptomatic infection in the F1 generation. Our findings demonstrate that congenital infection occurs during post-acute and chronic infection in dams. Development of pregnancy during acute babesiosis seems not to be possible. This suggests that *Babesia* infection may play a role in pregnancy losses in this experimental model. Further characterization of this model will contribute significantly to our understanding of the cellular and immunological mechanisms expressed during pregnancy in *Babesia-*infected females.
